# An Antisense RNA Fine-Tunes Gene Expression of the Type II MazEF Toxin-Antitoxin System

**DOI:** 10.1128/mbio.03443-21

**Published:** 2022-01-11

**Authors:** Taylor Van Gundy, Edward Martin, Jeremy Bono, Olivia Hatton, Meghan C. Lybecker

**Affiliations:** a Department of Biology, University of Colorado, Colorado Springs, Colorado, USA; b Department of Molecular Biology, Colorado College, Colorado Springs, Colorado, USA; Institut Pasteur

**Keywords:** MazEF, toxin-antitoxin systems, antisense RNA, pervasive transcription, regulatory RNA

## Abstract

Despite their ubiquitous nature, few antisense RNAs have been functionally characterized, and this class of RNAs is considered by some to be transcriptional noise. Here, we report that an antisense RNA (asRNA), aMEF (antisense *mazEF*), functions as a dual regulator for the type II toxin-antitoxin (TA) system *mazEF*. Unlike type I TA systems and many other regulatory asRNAs, aMEF stimulates the synthesis and translation of *mazEF* rather than inhibition and degradation. Our data indicate that a double-stranded RNA intermediate and RNase III are not necessary for aMEF-dependent regulation of *mazEF* expression. The lack of conservation of asRNA promoters has been used to support the hypothesis that asRNAs are spurious transcriptional noise and nonfunctional. We demonstrate that the aMEF promoter is active and functional in Escherichia coli despite poor sequence conservation, indicating that the lack of promoter sequence conservation should not be correlated with functionality.

## INTRODUCTION

Transcription was initially thought to adhere to gene boundaries and produce only mRNA, tRNA, and rRNA. However, pervasive transcription was identified with the advent of RNA sequencing, demonstrating that most of the genome is transcribed regardless of genomic context (1–3). In bacteria, most pervasive transcription occurs antisense to or within (intragenic) annotated genes. However, the function of pervasive transcription remains intensely debated in the RNA biology field. Several groups have shown that putative antisense RNA (asRNA) promoter sequences are not well conserved and therefore deem that they are likely not biologically functional (4, 5). Although sequence conservation is a useful metric to predict whether uncharacterized sequences are functional, sequence divergence does not necessarily indicate a lack of functional conservation. As recently reviewed by Georg and Hess, specific lowly conserved asRNAs are important regulators of gene expression (6). Many asRNA-dependent gene regulation mechanisms require the formation of a double-stranded RNA duplex from the base pairing of the sense RNAs (sRNAs) and asRNAs. However, several recent studies suggest that the act of transcription of asRNA is functional in DNA repair processes, gene regulation via transcriptional interference, and, potentially, functional RNA-DNA hybrid formation (6–14). The asRNA and transcription of the asRNA can regulate the gene expression of the sense mRNA.

Toxin-antitoxin (TA) systems are composed of a “toxin” that after activation either kills cells or confers growth stasis and an “antitoxin” that regulates the activity of the toxin (15). The type I toxin-antitoxin systems utilize asRNAs as antitoxins to regulate the expression of their cognate mRNA toxin. The asRNA binds the mRNA and can inhibit toxin translation or promote toxin mRNA degradation (15). In contrast, type II TA systems are composed of an antitoxin and a toxin that are both proteins (15). Unlike type I TA systems, type II TA systems do not have a reported asRNA-dependent gene regulation mechanism. The type II TA system MazEF has been extensively studied in bacteria and implicated in both programmed cell death (PCD) and persistence (16–18). *mazEF* in Escherichia coli encodes a labile antitoxin, MazE, and a stable toxin, MazF (16, 19). MazF is an endoribonuclease that cleaves RNA at ACA sites, which results in the inhibition of protein synthesis and an altered transcriptome (20). We identified an antisense RNA opposite the *mazEF* operon, which we have termed antisense *mazEF* (aMEF).

In the present study, we demonstrate that aMEF or its transcription regulates the expression of *mazEF*. A reverse genetic approach using directed deletions is not feasible with an asRNA without disturbing the overlapping coding sequence. Previously, most laboratories studied asRNA-dependent gene regulation by overexpressing the asRNA in *trans*. However, asRNAs are transcribed in *cis* opposite their sense mRNA counterpart. The overexpression of an asRNA in *trans* does not mimic the in *cis* expression of the asRNA and may not uncover the regulation of the mRNA by an asRNA. Indeed, even the transient overexpression of *trans*-acting sRNAs is wrought with numerous caveats (21). Moreover, producing a large amount of an sRNA may result in gene expression artifacts caused by competing with other sRNAs for binding with RNA chaperones, affecting the RNA chaperones’ availability in the cell (22–26). Here, we utilize a technique to knock down asRNA production endogenously to assay asRNA function (27). We identified the noncanonical aMEF promoter and precisely mutated it on the chromosome to knock down the levels of asRNA transcription without disrupting the open reading frame (ORF) of MazF. In addition, we show that the aMEF promoter is active and functional in E. coli despite relatively low levels of sequence conservation. These results demonstrate that the levels of sequence conservation do not always accurately predict the levels of functional conservation.

## RESULTS

### Construction of an aMEF promoter mutant strain.

**FIG 1:**
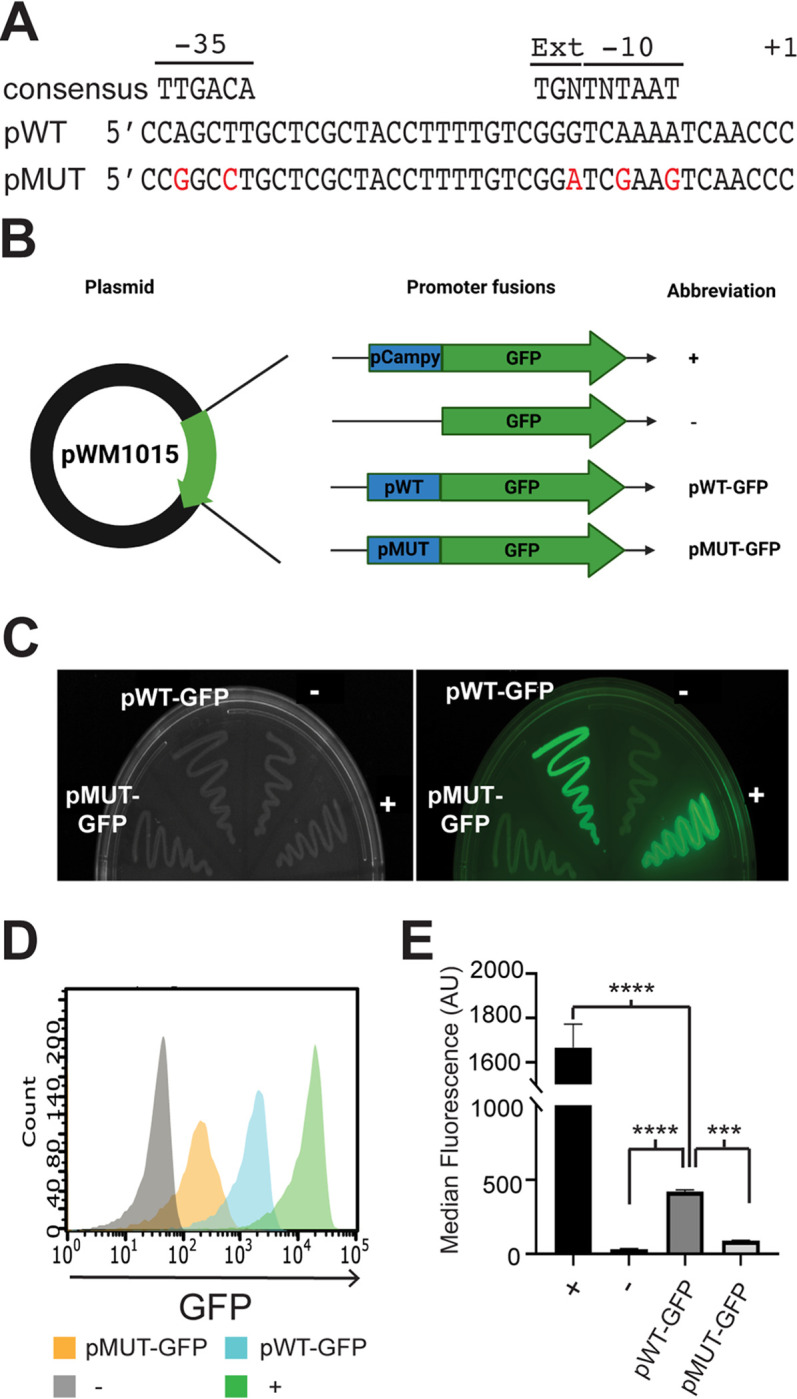
aMEF promoter characterization and mutagenesis. (A) Sequence of putative wild-type and mutant aMEF promoters. Silent mutations are in red. The σ^70^ consensus sequences (−10, extended −10, and −35 regions) and the TSS (+1) are denoted. (B) Illustration of the plasmids constructed for *gfp* promoter fusion assays. The pWM1015 plasmid was used as the vector for *gfp* promoter fusions. The promoters inserted include a constitutive promoter (consensus Campylobacter promoter) as the positive control (+), a promoterless reporter as the negative control (−), and the aMEF wild-type (pWT-GFP) and mutant (pMUT-GFP) promoters. (C) Fluorescence and white-light images of E. coli harboring the *gfp* transcriptional fusion plasmids. (D) Histogram overlay of *gfp* promoter fusion strains analyzed by flow cytometry. The positive control (+) is depicted in green, the negative control (−) is in gray, aMEF pWT-GFP is in blue, and aMEF pMUT-GFP is in orange. (E) Quantification of flow cytometry data using median fluorescence. Error bars represent standard errors of means (SEM). One-way analysis of variance (ANOVA) indicated differences among the means (*F* value, 204.9; *P* < 0.0001), which was followed up by Sidak’s multiple-comparison analysis for the positive versus the negative control (****, *P* < 0.0001 [Sidak’s multiple-comparison test]), the negative control versus pWT-GFP (****, *P* < 0.0001 [Sidak’s multiple-comparison test]), and pWT-GFP versus pMUT-GFP (**, *P* = 0.0014 [Sidak’s multiple-comparison test]). AU, arbitrary units.

We and others have identified an asRNA opposite the *mazEF* operon in transcriptomic data (28, 29). To elucidate the function of the asRNA, termed aMEF, we wanted to generate an aMEF deletion strain. However, traditional deletion mutagenesis could not be performed on aMEF because in addition to aMEF, a region of the *mazEF* genes would also be deleted. Therefore, we aimed to generate silent mutations in the promoter of aMEF to knock down the transcription of the asRNA while maintaining the MazEF ORFs on the chromosome. To this end, we first identified and characterized the activity of the asRNA promoter. A global 5′-end analysis identified a putative transcriptional start site (TSS) for aMEF (29). Notably, the sequence upstream of the aMEF TSS does not resemble a canonical σ^70^ promoter in the −10, −35, or extended −10 TGN regions (Fig. 1A). To validate and characterize the transcriptional activity of the aMEF putative promoter, we constructed green fluorescent protein (GFP) gene (*gfp*) transcriptional fusions in the plasmid pWM1015. The 75 bp upstream of the asRNA aMEF TSS was cloned into the *gfp* reporter plasmid pWM1015 (30). In addition, a constitutive promoter from Campylobacter was fused to *gfp* as a positive control, and a promoterless fusion was used as a negative control. *gfp* expression was visualized and quantified by fluorescence plate assays and flow cytometry (Fig. 1). The data show that the aMEF putative promoter (pWT-GFP) initiates significantly higher transcription than that of the promoterless negative control (pWM1015 empty [−]) albeit significantly lower than that of the positive control (pWM1015 [+]) (Fig. 1). Next, we designed five silent point mutations in the putative −10 and −35 regions of the aMEF promoter that maintained the ORF of the *mazF* gene but were predicted to disrupt aMEF promoter function (Fig. 1; see also Fig. S1 in the supplemental material). Flow cytometry showed that the aMEF mutant promoter (pMUT-GFP) activity is significantly lower than that of the wild-type aMEF promoter (pWT-GFP), reducing the transcriptional activity of the promoter (Fig. 1). Activated MazF cleaves its own mRNA at 7 ACA trinucleotide sites within the coding region of *mazF* in a negative feedback loop (17). The point mutations did not alter these cleavage sites or the ORF of MazF (Fig. S1A). Moreover, the predicted secondary structures of the wild-type and aMEF promoter mutant *mazF* mRNAs were modeled using the Vienna RNA package, which revealed that the point mutations do not drastically alter the structure (Fig. S1B).

10.1128/mbio.03443-21.1 FIG S1 Silent mutations do not affect *mazF* secondary structure. (A) Nucleotide and amino sequences of wild-type and mutant aMEF, pWT and pMUT, respectively. The silent mutations are shown in red. (B) Vienna RNA package folding of wild-type and mutant aMEF *mazF* mRNAs. Arrowheads denote the nucleotides that were mutated. Download FIG S1, TIF file, 1.4 MB. Copyright © 2022 Van Gundy et al. 2022 Van Gundy et al. https://creativecommons.org/licenses/by/4.0/ This content is distributed under the terms of the Creative Commons Attribution 4.0 International license.

**FIG 2:**
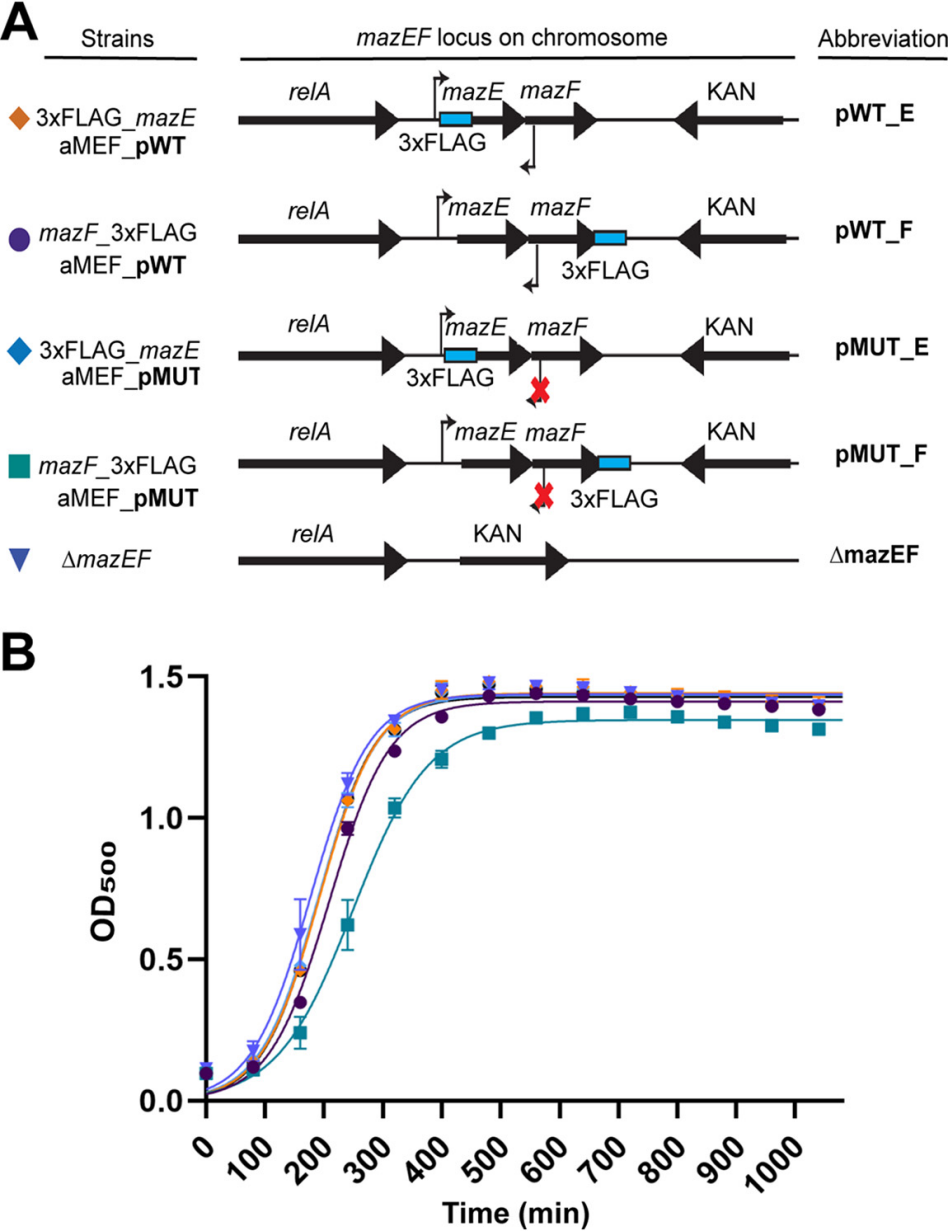
Construction and validation of the aMEF mutant and epitope-tagged MazE and MazF strains. (A) Illustration of strains generated for this study. All mutations, deletions, and epitope tags were recombined into the *mazEF* locus on the chromosome. (B) Growth curve of the *mazEF* strains depicted in panel A.

To determine the role of aMEF in *mazEF* gene regulation, we mutated the aMEF promoter on the chromosome to generate an aMEF knockdown strain. The five silent mutations characterized in the transcriptional fusions (Fig. 1) were moved into the *mazEF* locus on the chromosome along with C-terminally 3×FLAG-tagged MazF and N-terminally 3×FLAG-tagged MazE; the strains were termed pMUT_F and pMUT_E, respectively (Fig. 2A). In addition, MazF and MazE 3×FLAG-tagged strains were generated with a wild-type aMEF promoter and termed pWT_F and pWT_E, respectively. Finally, as a control, we generated a *mazEF* deletion strain (Δ*mazEF*) (Fig. 2A). Next, we verified that the silent mutations and the 3×FLAG tags did not disrupt the activity, translation, or degradation of MazE or MazF. The deletion of *mazE* or the artificial overexpression of *mazF* leads to a decreased growth rate or cell death, respectively (31, 32). Therefore, we assayed the growth rates of the parental and aMEF pWT_E and pMUT_E 3×FLAG-tagged strains to assay MazE function. All strains grew similarly, indicating that the silent mutations and the 3×FLAG tags did not alter the activity of MazE (Fig. 2B). To confirm that the silent mutations and the 3×FLAG tag did not alter MazF activity or its ability to be translated, we induced and overexpressed wild-type MazF and MazF 3×FLAG-tagged aMEF pWT and pMUT from the pD441 plasmid. Growth curve assays were performed by growing cells to the mid-logarithmic growth phase and then inducing cells with isopropyl-β-d-thiogalactopyranoside (IPTG). The growth curve demonstrates cessation of cell growth by all overexpressed MazF proteins (Fig. S2). Immunoblot analyses show that after induction, MazF protein levels are similar regardless of whether the aMEF promoter is mutated or not (pWT versus pMUT), indicating that the silent mutations did not affect MazF translation (Fig. S2C). Finally, we verified that the N-terminal 3×FLAG tag on the chromosomal *mazE* gene did not affect its degradation. MazE is degraded by Lon and Clp proteases upon environmental stresses, including osmotic shock (31, 33–35). Immunoblot assays demonstrate that the levels of 3×FLAG-tagged MazE are reduced after osmotic shock, indicating its degradation is not affected by the 3×FLAG tag (Fig. S2D). Taken together, our data demonstrate that the 3×FLAG tags and the silent mutations do not affect the degradation or activity of MazE_FLAG or the translation and activity of MazF_FLAG.

10.1128/mbio.03443-21.2 FIG S2 FLAG tagging and silent mutations do not affect MazEF activity, translation, or degradation. (A) Illustration of the plasmids constructed for the overexpression of wild-type and 3×FLAG-tagged pWT and pMUT MazF. (B) Growth curves of the strains depicted in panel A after induction with IPTG at an OD_600_ of 0.3. Black dots indicate E. coli wild-type parent strain BW25113. (C) Immunoblot analyses of whole-cell lysates from strains harboring overexpression plasmids depicted in panel A. Samples were taken before IPTG induction and 60 min after induction. Immunoblots were probed with M2 anti-FLAG antibody and anti-GroEL antibody. Three independent biological replicates were performed, and representative data are shown. Error bars represent standard errors of means (SEM). Two-way repeated measures (RM) ANOVA indicated differences among the means for time versus strain (two-way RM ANOVA *F* value of 0.4977; ns, not significant [*P* value of 0.5194]) and strain versus strain (two-way RM ANOVA *F* value of 1.407; ns, not significant [*P* value of 0.3013]). (D) Immunoblot analyses of whole-cell lysates from pWT and pMUT MazE FLAG-tagged strains in the presence (+) or absence (−) of 0.9 M NaCl. Immunoblots were probed with M2 anti-FLAG antibody and anti-GroEL. Three independent biological replicates were performed, and representative data are shown. Download FIG S2, TIF file, 1.9 MB. Copyright © 2022 Van Gundy et al. 2022 Van Gundy et al. https://creativecommons.org/licenses/by/4.0/ This content is distributed under the terms of the Creative Commons Attribution 4.0 International license.

### aMEF transcription regulates *mazEF* expression.

**FIG 3:**
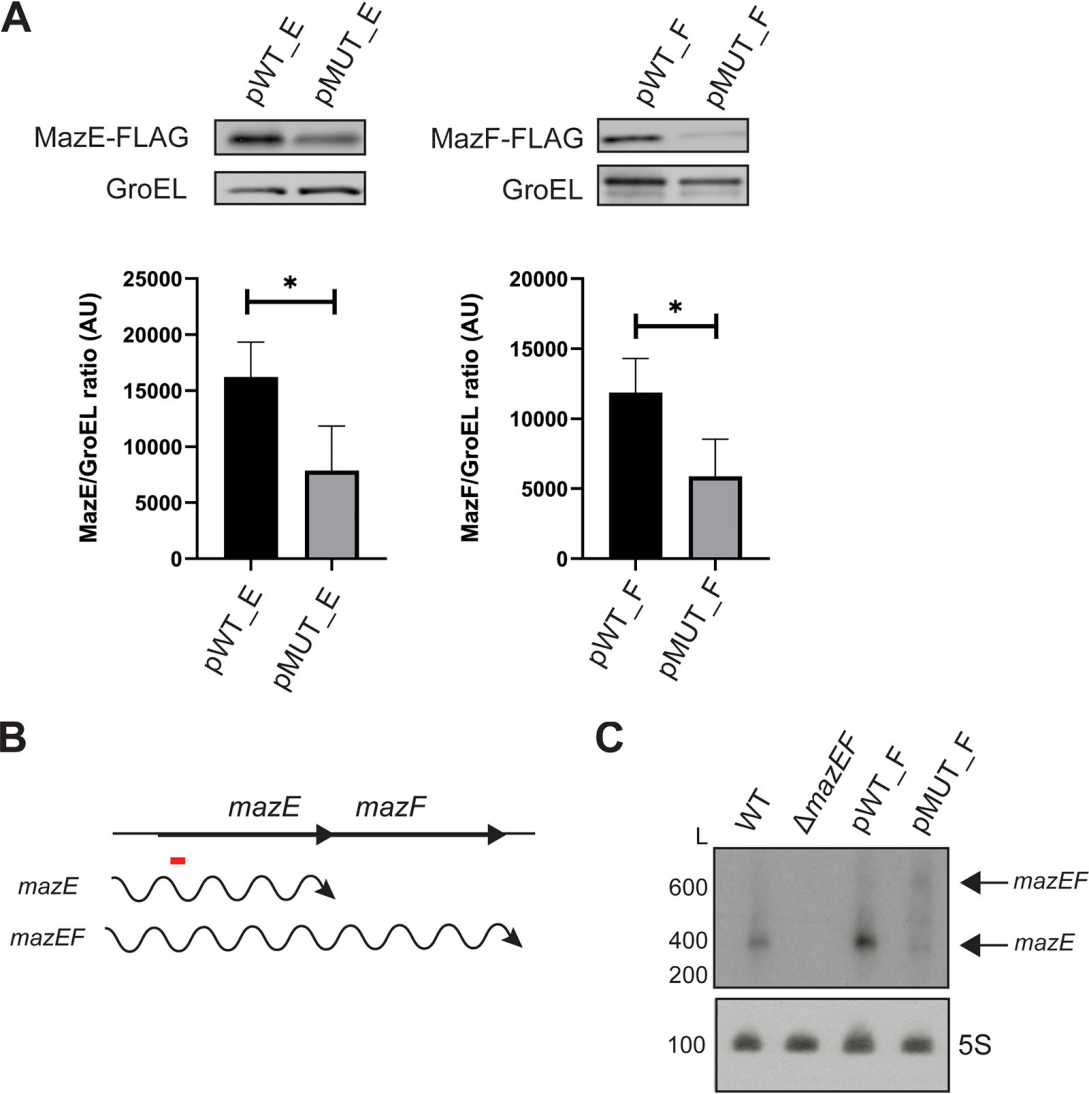
aMEF regulates *mazEF* expression at the logarithmic growth phase. (A) Immunoblot analyses of whole-cell lysates of wild-type (pWT) and mutant (pMUT) aMEF *mazE* and *mazF* FLAG-tagged strains. Immunoblots were probed with M2 anti-FLAG antibody and anti-GroEL antibody. Three independent biological replicates were performed, and representative data are shown. Immunoblot signals were quantified by ImageJ and analyzed via GraphPad Prism for MazE (Welch’s *t* test *t* = 2.848; *, *P* = 0.0465) and MazF (Welch’s *t* test *t* = 4.571; *, *P* = 0.0121). (B) Diagram illustrating the genomic context and sizes of transcripts based on Northern blot analysis. The red bar represents the location of the oligonucleotide probe used for Northern blotting of *mazEF*. The wavy lines represent the transcripts detected in the Northern blot. (C) Northern blot analysis of total RNAs isolated from the pWT_F, pMUT_F, Δ*mazEF*, and isogenic wild-type (WT) strains fractionated on a 6% denaturing polyacrylamide gel, blotted onto a nylon membrane, and hybridized with a radioactively labeled oligonucleotide probe for *mazEF*. 5S RNA was probed as the loading control. The RNA ladder (L) size in nucleotides is indicated. Three independent biological replicates were performed, and representative data are shown.

We hypothesized that the asRNA aMEF regulates the expression of its sense counterpart *mazEF*. We utilized the 3×FLAG strains to examine MazE and MazF levels in the aMEF promoter mutant (pMUT) strains at the logarithmic growth phase. Immunoblot analyses demonstrate that both MazE_FLAG and MazF_FLAG levels are reduced in the aMEF promoter mutant strains (pMUT) compared to the aMEF promoter wild-type (pWT) strains (Fig. 3A), suggesting that the aMEF transcript and/or transcription stimulates the expression of MazE and MazF. To determine if aMEF regulates steady-state levels of the *mazEF* transcript at the logarithmic growth phase, we performed Northern blot analysis (Fig. 3B and C). Engelberg-Kulka et al. initially reported that *mazEF* are cotranscribed (16). More recently, Gross et al. overexpressed the operon from an inducible promoter and demonstrated that in addition to the cotranscribed *mazEF*, a smaller *mazE* transcript was also produced (36). Northern blotting revealed that the predominant endogenous transcript is approximately 400 nucleotides (nt), corresponding to the size of a *mazE* transcript only. There is also a less abundant transcript detected predominantly in the pMUT strain at approximately 600 nt that corresponds to the size of the cotranscribed *mazEF* transcript. In the aMEF pMUT strain, there is less steady-state *mazE* transcript and more *mazEF* dicistronic transcript than in the aMEF pWT strain (Fig. 3C). None of the *mazEF* transcripts were detected in the Δ*mazEF* strain, confirming that the bands detected are specific to the *mazEF* transcripts. The aMEF transcript was not detectable by Northern blotting in the parent, pWT, or pMUT strain.

### aMEF regulation of MazEF is not dependent on RNase III.

**FIG 4:**
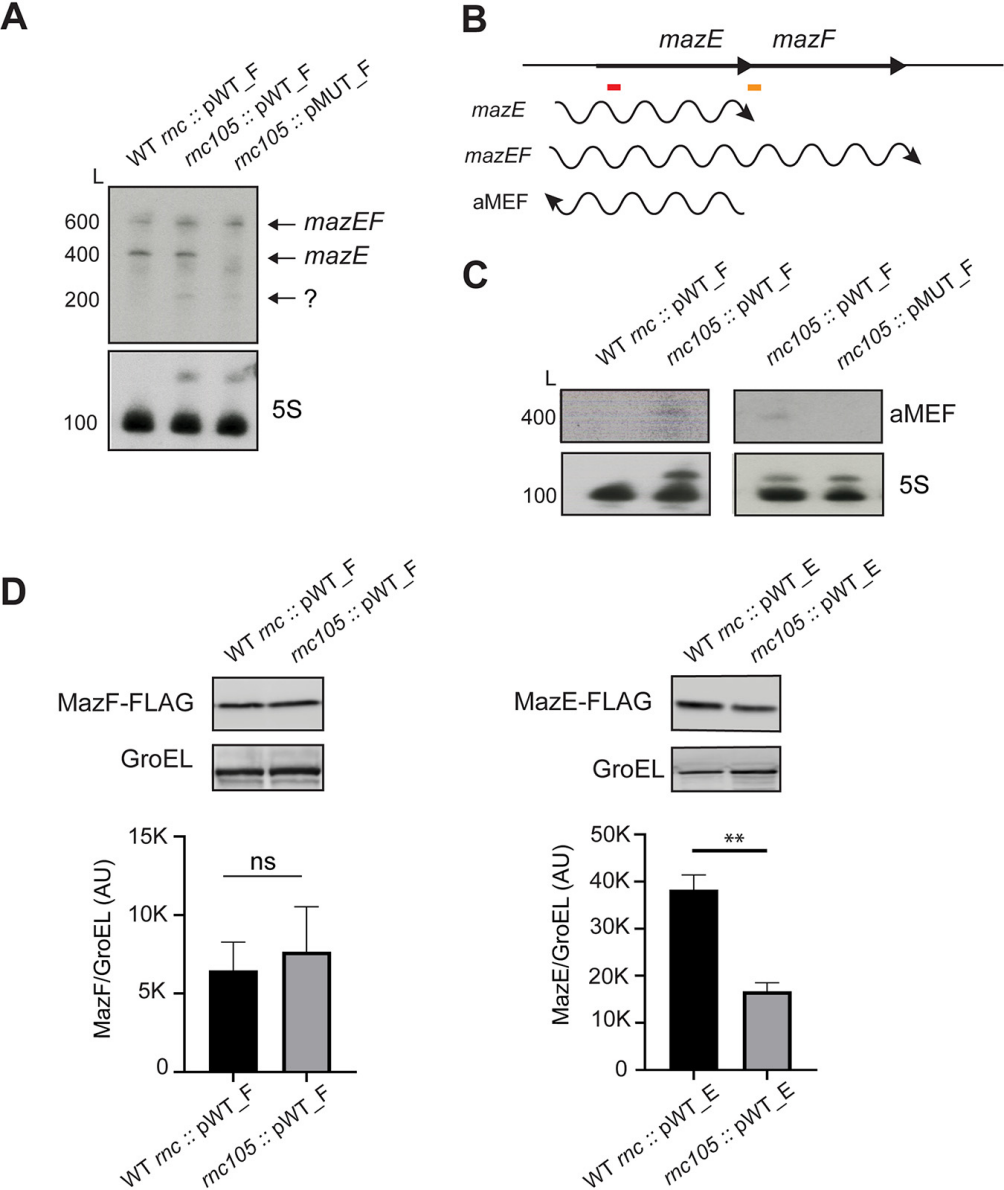
aMEF regulation of *mazEF* is not RNase III dependent. (A) Northern blot analysis of total RNA isolated from the WT *rnc*::pWT_F, *rnc*105::pWT_F, or *rnc*105::pMUT_F strain as described in the Fig. 3 legend. 5S RNA was probed as a loading control. RNase III processes the 5S rRNA from a 9S precursor (64). In an RNase III mutant strain, unprocessed 9S rRNA is detected in addition to the 5S RNA. (B) Diagram illustrating the genomic context and sizes of transcripts based on Northern blot analyses. The red and orange bars represent the locations of oligonucleotide probes used for Northern blotting of *mazEF* and aMEF, respectively. The wavy lines represent the transcripts detected in the Northern blot. (C) Northern blot analyses of total RNA isolated from the WT *rnc*::pWT_F, *rnc*105::pWT_F, or *rnc*105::pMUT_F strain probed for aMEF as described in the Fig. 3 legend. 5S RNA was probed as a loading control. (D) Immunoblot analyses of MazE_FLAG and MazF_FLAG in RNase III wild-type (WT *rnc*) or cleavage mutant (*rnc*105) strains probed with M2 anti-FLAG and anti-GroEL antibodies. Immunoblot signals were quantified by ImageJ and analyzed via GraphPad Prism for MazE pWT_E (for WT *rnc* versus *rnc*105, Welch’s *t* test *t* = 10.21 [**, *P* = 0.0014]) and MazF pWT_F (for WT *rnc* versus *rnc*105, Welch’s *t* test *t* = 0.6149 [ns, not significant {*P* = 0.5776}]).

Many characterized antisense RNAs regulate gene expression by binding to their complementary sense mRNAs, forming a double-stranded RNA (dsRNA). RNase III is an endoribonuclease that specifically cleaves dsRNA, which can either destabilize or stabilize the RNAs. Recently, we globally identified dsRNA regions formed from asRNA-mRNA duplexes in E. coli (28). We did not identify a dsRNA region in *mazEF* in our transcriptomic data, suggesting that a dsRNA does not form between aMEF and *mazEF*. Furthermore, no RNase III cleavage sites were mapped in *mazEF* or aMEF in two separate studies that globally mapped RNase III cleavage sites in E. coli (37, 38). We hypothesized that aMEF-dependent *mazEF* regulation is not dsRNA dependent and should not be regulated by RNase III. To test this hypothesis, we characterized the steady-state levels of *mazE* and *mazEF* in an RNase III cleavage mutant strain (*rnc*105) and its parent strain (WT *rnc*) (39). In addition, we mutated the aMEF promoter and inserted 3×FLAG-tagged MazE and -F into the *mazEF* locus in the RNase III mutant and the parent strain, generating a double mutant strain (*rnc*105::pMUT_F and *rnc*105::pMUT_E) and a control strain (WT *rnc*::pMUT_E and WT *rnc*::pMUT_F). We also inserted 3×FLAG-tagged MazE and MazF with the wild-type aMEF promoter into the RNase III mutant and the parent strain (WT *rnc*::pWT_F, WT *rnc*::pWT_E, *rnc*105::pWT_F, and *rnc*105::pWT_E). Northern blot analysis demonstrates that neither the dicistronic *mazEF* nor the *mazE* steady-state levels were affected by the loss of RNase III cleavage (Fig. 4A). In addition, the *mazE* transcript was still produced in the RNase III cleavage mutant strain, indicating that RNase III is not responsible for cleaving and processing the *mazE* transcript (Fig. 4A). Notably, a smaller, approximately 200-nt transcript was detected in the RNase III cleavage mutant strains. We postulate that this transcript is a result of the early termination of the *mazEF* transcript or processing by a different RNase. RNase III is a pleiotropic regulator that affects the gene expression of approximately 10% of the genome and has cleavage sites in genes that affect transcription elongation/termination (NusG and Rho leader region) and RNase E (37, 40). The aMEF pMUT/RNase III cleavage double mutant strains did not produce the *mazE* transcript as seen in the pMUT BW25113 strain (Fig. 3). Furthermore, MazE_FLAG and MazF_FLAG protein levels were also decreased in the RNase III cleavage and aMEF promoter double mutant strains (*rnc*105::pMUT) compared to the RNase III cleavage mutant and aMEF promoter wild-type strains (*rnc*105::pWT) (Fig. S3). These data are consistent with the aMEF promoter mutant phenotype observed in the BW25113 E. coli strain (Fig. 3). Taken together, these data suggest that RNase III does not play a role in the aMEF-dependent production of the monocistronic *mazE* transcript or the aMEF-dependent regulation of *mazEF*.

10.1128/mbio.03443-21.3 FIG S3 aMEF regulates *mazEF* expression in an RNase III mutant strain. (A) Immunoblot analyses of whole-cell lysates of RNase III cleavage mutant (*rnc*105) strains that also have aMEF wild-type and mutant promoters and *mazE* and *mazF* 3×FLAG tagged at the *mazEF* loci (pWT_E, pWT_F, pMUT_E, and pMUT_F). Immunoblots were probed with M2 anti-FLAG antibody and anti-GroEL antibody. Three independent biological replicates were performed, and representative data are shown. Immunoblot signals were quantified by ImageJ and analyzed via GraphPad Prism for MazE *rnc*105::pWT_E versus pMUT_E (Welch’s *t* test *t* = 4.089; *, *P* = 0.0428) and MazF *rnc*105::pWT_F versus pMUT_F (Welch’s *t* test *t* = 2.986; *, *P* = 0.0413). Download FIG S3, TIF file, 1.3 MB. Copyright © 2022 Van Gundy et al. 2022 Van Gundy et al. https://creativecommons.org/licenses/by/4.0/ This content is distributed under the terms of the Creative Commons Attribution 4.0 International license.

We and others have shown that many asRNAs are undetectable in wild-type cells because they are degraded by RNases, including RNase III (28, 37, 41). Therefore, many asRNAs can be detected only in RNase mutant strains. We were unable to detect an aMEF transcript via Northern blotting in the wild-type strain but detected a 400-nt transcript in the RNase III mutant strain, suggesting that aMEF is degraded by RNase III (Fig. 4C). In addition, aMEF was undetectable in the RNase III pMUT double mutant strain, validating the loss of aMEF in the pMUT strains (Fig. 4C).

Our data demonstrate that RNase III does not regulate *mazE* or *mazEF* steady-state transcript levels (Fig. 4A); therefore, we hypothesized that it would not regulate protein levels either. Immunoblot analyses revealed that MazF_FLAG levels were similar in the wild-type and RNase III cleavage mutant strains, but surprisingly, MazE_FLAG levels were slightly downregulated in the RNase III mutant strain (Fig. 4D). Taken together, these data suggest that the slight decrease in MazE levels detected in the RNase III mutant strain occurs posttranscriptionally by either a decrease in translation or an increase in proteolysis.

### aMEF-dependent regulation of MazEF affects *dps* expression.

**FIG 5:**
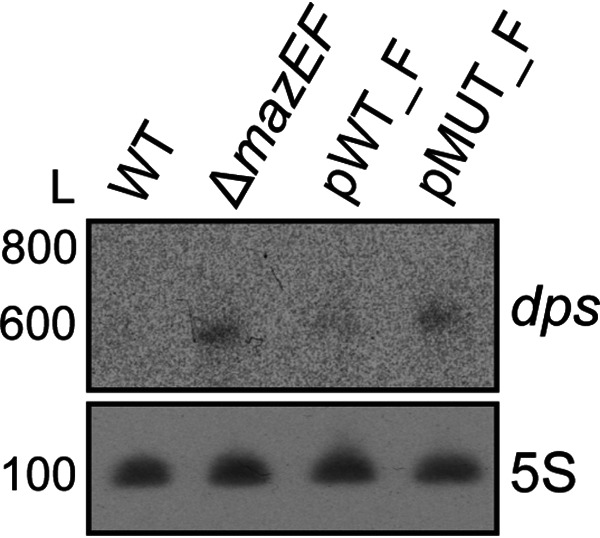
MazEF regulates *dps* expression at logarithmic growth phase. Northern blot analysis of *dps* was performed. RNA was isolated from the wild-type, Δ*mazEF*, pWT, and pMUT strains grown in LB to an OD_600_ of ∼0.4 to 0.6, fractionated on a denaturing gel, blotted onto a membrane, and probed with a radiolabeled oligonucleotide as described in the Fig. 3 legend. 5S RNA was probed as a loading control.

Our data demonstrate that a decrease in aMEF transcription results in significantly lower levels of MazE_FLAG and MazF_FLAG proteins. Both proteins are reduced by ∼2-fold in the pMUT strains (Fig. 3). We hypothesize that the decrease in the expression of *mazEF* in the pMUT strains would affect the ability of MazEF to carry out its biological function. However, the biological function of MazEF remains undefined (20, 42–45). Recently, a study globally identified proteins, including the DNA-binding protein from starved cells (Dps), that were upregulated in a wild-type strain compared to a *mazEF* deletion strain after DNA damage (42). Dps is a ferritin that interacts with and condenses DNA in the bacterial nucleoid during starvation. Dps levels are increased in response to entry into stationary phase and oxidative stress but are relatively low at logarithmic phase (46). Our data demonstrate that aMEF regulates MazF levels at the logarithmic growth phase, and we hypothesized that MazF may regulate Dps at the logarithmic growth phase. Northern blot data reveal higher levels of the *dps* transcript in the pMUT_F and Δ*mazEF* mutant strains than in the wild-type strains (Fig. 5). These data suggest that the MazEF TA system represses the expression of Dps at the logarithmic growth phase and that aMEF significantly affects the biological levels of MazEF.

### The putative aMEF promoters from diverse genera of bacteria are active in E. coli.

**FIG 6:**
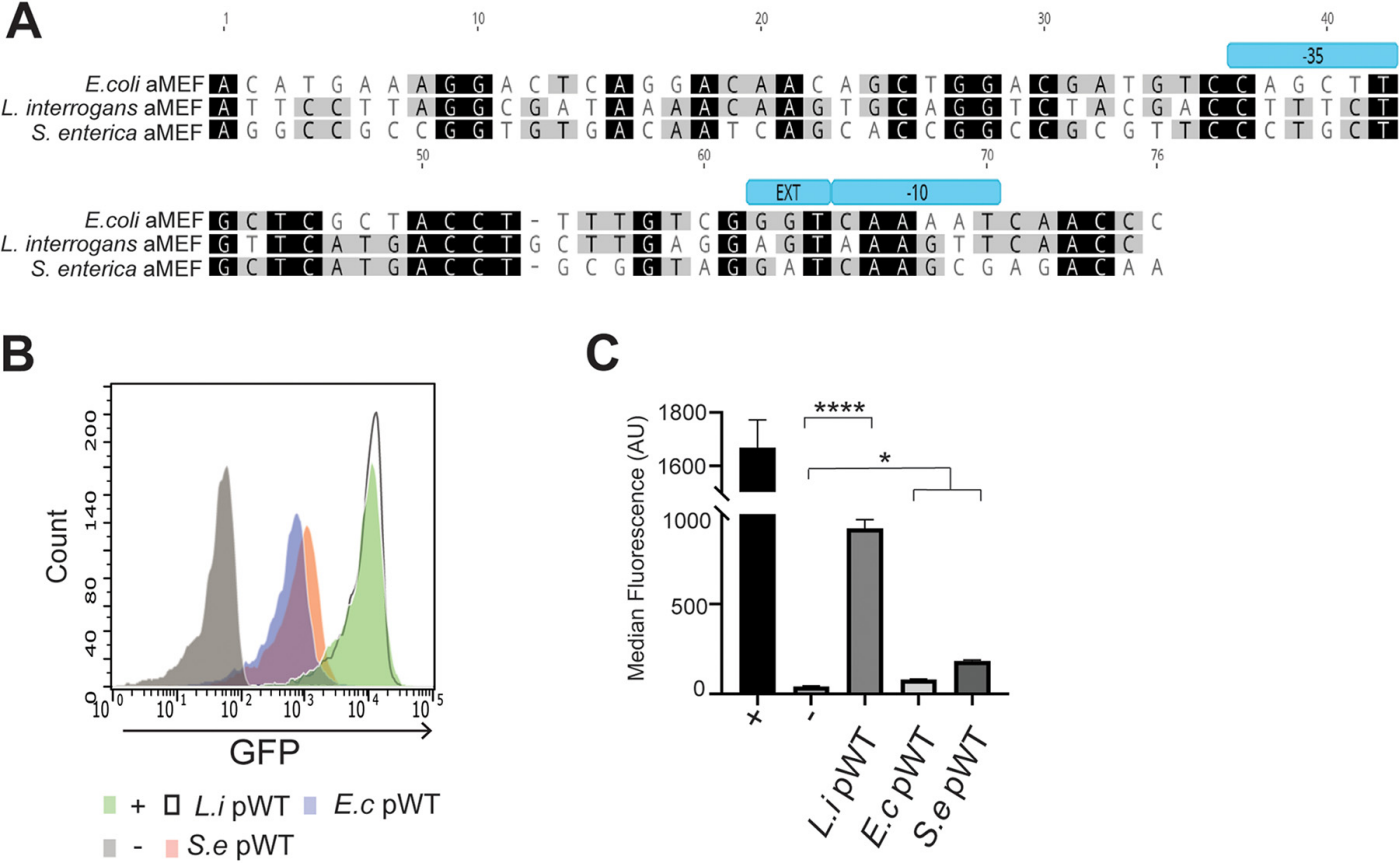
Putative aMEF promoters from diverse genera of bacteria are active in E. coli. (A) Pairwise alignment of the putative aMEF promoter sequences from E. coli (row 1), L. interrogans (row 2), and S. enterica (row 3). (B) Histogram overlay of *gfp* promoter fusions in E. coli analyzed by flow cytometry. The positive control (+) is in green, the negative control (−) is in gray, the aMEF promoter from E. coli (*E.c*) is in purple, that from S. enterica (*S.e*) is in orange, and that from L. interrogans (*L.i*) is shown as an unfilled, black solid line. (C) Quantification of flow cytometry data using median fluorescence. Error bars represent standard errors of means (SEM). One-way ANOVA indicated differences among the means (*F* = 131.1; *P* < 0.0001), which was followed by Sidak’s multiple-comparison analysis for all three test groups versus the negative control: the negative control versus L. interrogans pWT (****, *P* < 0.0001), the negative control versus E. coli pWT (*, *P* < 0.0345), and the negative control versus S. enterica (*, *P* < 0.0295).

In this study, we demonstrate that an asRNA, aMEF, is functional and required for the expression of MazEF in E. coli. MazEF protein sequences are conserved and found in many different bacteria. However, the DNA sequences of the toxins and the putative antisense promoter regions are less conserved (47). Several groups have reported that the lack of sequence conservation among antisense promoters suggests that they are not functional and are the result of “happenstance” or spurious transcription (4, 5). We hypothesized that despite the relatively low levels of sequence conservation among DNA sequences of MazEF between bacterial species, the putative aMEF promoters would be active (47, 48). We chose to test the aMEF putative promoters from Salmonella enterica, a close relative of E. coli, and Leptospira interrogans, a divergent spirochete (Fig. 6A). We generated *gfp* transcriptional fusions and tested their promoter activity in E. coli. Putative aMEF promoters from S. enterica and L. interrogans were fused to *gfp* in the pWM1015 plasmid, and transcriptional activity was quantified by flow cytometry. aMEF promoters from L. interrogans and S. enterica were active in E. coli (Fig. 6B and C). RNA polymerase (RNAP) can initiate transcription from diverse DNA sequences, resulting in genome-wide transcription, termed pervasive transcription (3, 49). aMEF promoter activity is low, and steady-state levels are undetectable by Northern blotting in wild-type cells. The aMEF promoter sequence does not resemble the canonical σ^70^ sequence (Fig. 1A) and is poorly conserved (Fig. 6A) (47, 48). Taken together, these characteristics suggest that aMEF is a functional yet spurious transcript.

## DISCUSSION

**FIG 7:**
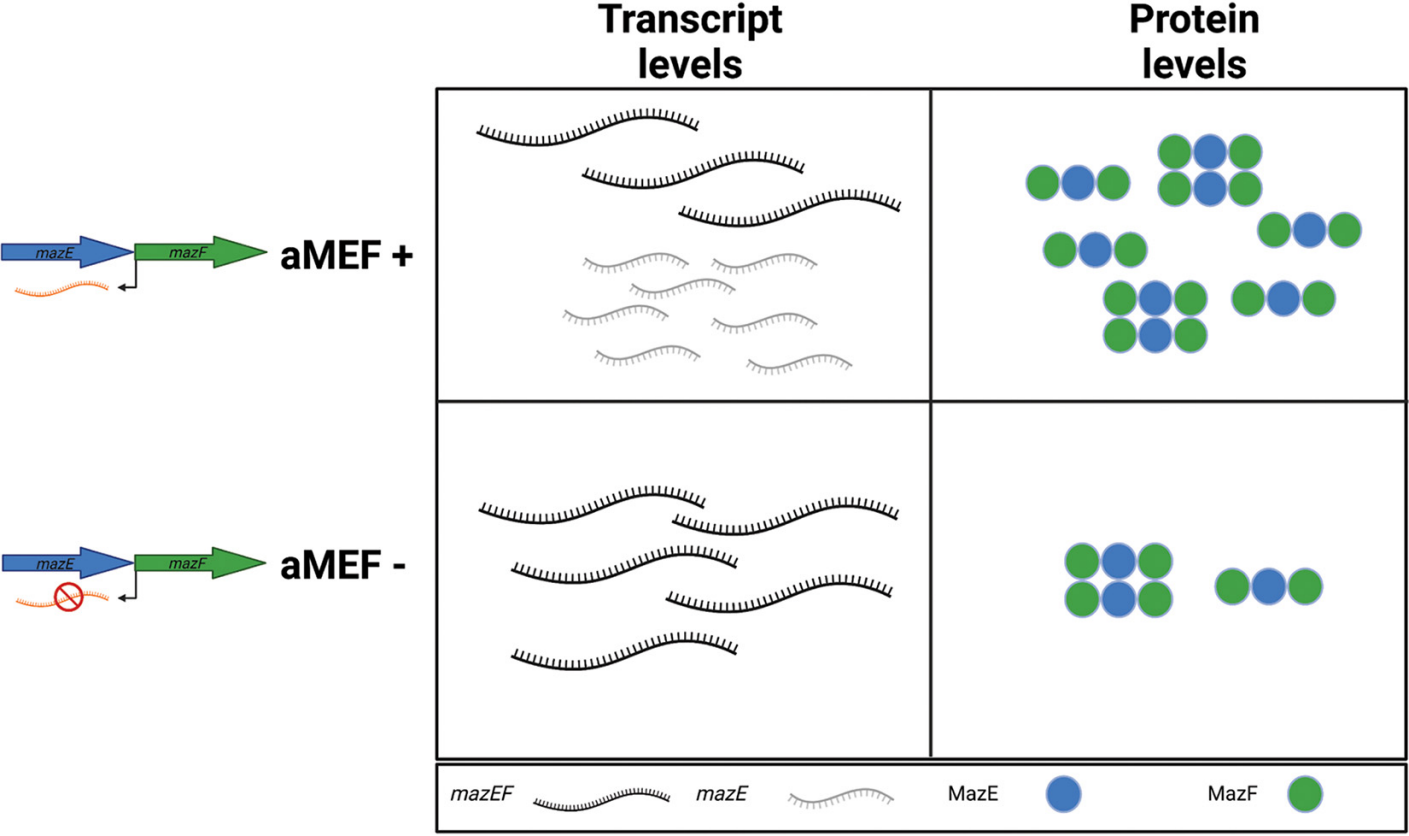
aMEF is a dual regulator that affects *mazEF* transcription and translation. The illustration summarizes the differences in *mazEF* transcript and protein levels in an aMEF mutant strain compared to a wild-type strain. The monocistronic *mazE* transcript is the predominant transcript in the wild-type strain but is nearly undetectable in the aMEF pMUT strain. In contrast, the dicistronic *mazEF* transcript is the predominant transcript in the aMEF pMUT strain and lowly expressed in the wild-type strain. In addition, both MazE and MazF protein levels are reduced in the aMEF pMUT strain compared to the wild-type strain despite there being more dicistronic *mazEF* transcript present in the pMUT strain.

Antisense RNAs are ubiquitously present across all domains of life, with various estimates on their abundance (2). The biological roles of most reported asRNAs remain unknown, and several groups propose that asRNAs are spurious nonfunctional transcripts (4, 5). The unbiased elucidation of the function of many asRNAs has been impeded since applying reverse genetic approaches to generate null mutants has been challenging. We circumvented these issues by introducing silent mutations into the promoter region of the asRNA, effectively decreasing its transcriptional activity while maintaining the ORF of its cognate gene. We demonstrate that the *mazEF* operon is regulated by the asRNA that we termed aMEF as part of a multifaceted regulatory pathway. aMEF is a dual-regulatory RNA that is both required to produce the *mazE* transcript and necessary for the synthesis of both MazE and MazF, unlike the asRNAs known to regulate type I TA systems (Fig. 7). asRNAs regulate type I toxins by inhibiting toxin transcription and/or translation through the direct binding of the asRNA to the toxin mRNA (15).

### RNase III is not necessary for aMEF-dependent *mazEF* regulation.

Our data demonstrate that RNase III does not play a role in the asRNA-dependent regulation of *mazEF* expression. In an RNase III cleavage mutant strain, the *mazE* transcript is still produced, and steady-state *mazEF* transcript levels are similar to those of the isogenic parent strain. In addition, MazF levels are unchanged in the RNase III mutant compared to the wild-type strain. However, MazE levels are slightly decreased in the RNase III mutant strain compared to a wild-type strain, but its steady-state transcript levels are not different. These data suggest that MazE translation or degradation may be regulated by RNase III. MazE is a labile protein that is degraded by both Lon and Clp proteases (31, 33, 35). Several RNase III cleavage sites in the ORFs of ClpA and ClpX have been identified (37). We postulate that in an RNase III cleavage mutant strain, the *clp* transcripts are not cleaved and degraded, leading to higher protein levels and increased degradation of MazE. While the steady-state levels of the *mazEF* transcripts are not affected by RNase III, the aMEF transcript was detectable by Northern blotting only in the RNase III cleavage mutant strain, suggesting that RNase III degrades aMEF. The RNase III-dependent degradation of aMEF may be due to intramolecular dsRNA regions in aMEF or because of duplexed aMEF and *mazEF* transcripts, but this degradation does not influence *mazEF* expression.

### aMEF regulates the production of the *mazE* transcript.

The *mazEF* operon was previously shown to be cotranscribed along with additional transcripts, including a *mazE* monocistronic transcript (36). However, the transcripts were detected only when overexpressed. We observed endogenous levels of *mazEF* transcripts expressed from the chromosome, and our Northern blot assays detected the presence of both the cotranscribed *mazEF* and monocistronic *mazE* transcripts. We did not reproducibly detect a *mazEFG* transcript (based on the predicted size) by Northern blotting (36). Monocistronic *mazE* is the most abundant transcript detected in the wild-type BW25113 strain under the growth conditions that we tested, while in the RNase III cleavage mutant strain and its isogenic parent, the dicistronic and monocistronic transcripts are more equally expressed. We postulate that this may be due to strain-to-strain variation. Regardless, all aMEF promoter mutant strains, whether in BW25113 or the RNase III strains, demonstrate the same *mazEF* misregulation. Other groups have reported that the transcription of *mazEF* is RelA dependent and inhibited by the production of (p)ppGpp under stressful conditions (50). Further experiments need to be performed to determine if aMEF contributes to the regulation of *mazEF* under these conditions.

Our data demonstrate that aMEF is responsible for the production of the *mazE* transcript, which could be a result of transcription termination or processing of the cotranscribed *mazEF* transcript (Fig. 3). As recently reviewed by Roberts, there are three known methods of transcription termination in E. coli: Rho-independent (intrinsic), Rho-dependent, and Mfd-dependent (DNA translocase) termination (51). We hypothesize that the production of the monocistronic *mazE* transcript is not dependent on Rho because the *mazEF* transcript was not differentially expressed upon Rho inhibition (52). Moreover, the 3′ end of the *mazE* transcript does not have the typical C-rich sequence normally utilized by Rho; however, there are alternative mechanisms for Rho to terminate transcription (52–54). Instead, we propose that the presence of RNAP on the aMEF promoter may block and stall RNAP actively transcribing *mazE*, allowing Rho-independent transcription termination. In addition, aMEF could terminate the transcription of *mazE* by binding to the nascent *mazE* transcript as it is being transcribed, leading to a structural change that results in the formation of a terminator stem-loop and the transcriptional termination of *mazE* (see Fig. S4 in the supplemental material). The asRNA RnaG is able to bind *icsA* mRNA in Shigella flexneri, which prevents the formation of an antitermination stem-loop, resulting in transcription termination (55). In addition, in Vibrio anguillarum, the asRNA RNAβ binds to the actively transcribing polycistronic *fatDCBA* mRNA and causes the termination of transcription near the stop codon of *fatA* (56, 57). Our data show that the aMEF transcript is lowly expressed and not detectable in wild-type strains, suggesting that the aMEF transcript itself is not needed for the production of the *mazE* monocistronic transcript or *mazEF* regulation. Alternatively, the *mazE* transcript could be generated by an endoribonuclease cleaving and processing the cotranscribed *mazEF* in an aMEF-dependent manner. We show that the likely candidate, RNase III, is not required for producing *mazE*, but another endoribonuclease could be responsible for the cleavage. Further studies need to be performed to elucidate the aMEF-dependent molecular mechanism that produces the *mazE* transcript.

10.1128/mbio.03443-21.4 FIG S4 Models of aMEF-dependent production of the *mazE* transcript. (A) Illustration of the *mazEF* genomic context and figure key. (B) A collision between two convergently transcribing RNA polymerases induces Rho-independent termination of transcription, resulting in the *mazE* monocistronic transcript (pink wavy line). (C) aMEF (black transcript) binding to the nascent *mazE* transcript induces transcriptional termination. Download FIG S4, TIF file, 1.5 MB. Copyright © 2022 Van Gundy et al. 2022 Van Gundy et al. https://creativecommons.org/licenses/by/4.0/ This content is distributed under the terms of the Creative Commons Attribution 4.0 International license.

### aMEF regulates the synthesis of MazE and MazF.

A reduction of both MazE and MazF proteins is detected in the aMEF pMUT strains compared to the aMEF pWT strains, which indicates that aMEF is required for the synthesis of MazE and MazF proteins. In the absence of aMEF, the *mazE* transcript is not produced, which could contribute to lower levels of MazE. However, there is more cotranscribed *mazEF* transcript in the aMEF pMUT_F strain than in the wild type but lower levels of MazF_FLAG protein, suggesting that aMEF regulates the translation of MazF. Cotranslation of MazEF has been proposed although not experimentally determined (16). In this case, MazF translation would require a −1 frameshift of the ribosome. A typical −1 frameshift requires a slippery sequence, also known as an X_XXY_YYZ motif (three identical nucleotides in a row and then a triple U or A, followed by an A, C, or U) (58). No such motif is found upstream of the start codon of *mazF*. Therefore, we propose that the clash of the RNAP transcribing *mazEF* and aMEF could cause the ribosome to stall at the start of the MazF ORF, facilitating the −1 frameshift (Fig. S5). Our data show that in the absence of aMEF transcription, more dicistronic *mazEF* transcript is produced (Fig. 3C). However, this transcript may be translationally inactive since there are lower levels of both MazE_FLAG and MazF_FLAG proteins in the pMUT strains than in the pWT strains (Fig. 3A). Translation inhibition could also occur via ribosome-binding-site (RBS) occlusion at the 5′ untranslated region (UTR) of the dicistronic transcript. The binding of aMEF could release the RBS and allow the translation of both proteins (Fig. S5). Further experimentation is needed to determine the molecular mechanism of aMEF-dependent *mazEF* regulation.

10.1128/mbio.03443-21.5 FIG S5 Models of aMEF-dependent MazE and MazF protein synthesis. (A) Convergently transcribing RNA polymerases collide, causing transcription and translation to pause and resulting in a −1 frameshift needed to translate *mazF*. (B) The nascent *mazEF* transcript (green wavy line) folds into a structure that occludes the Shine-Dalgarno sequence and inhibits translation initiation. aMEF (black transcript) binding to the *mazEF* transcript changes the structure and releases the ribosome-binding site, allowing translation. Download FIG S5, TIF file, 2.1 MB. Copyright © 2022 Van Gundy et al. 2022 Van Gundy et al. https://creativecommons.org/licenses/by/4.0/ This content is distributed under the terms of the Creative Commons Attribution 4.0 International license.

### aMEF-dependent regulation of MazEF affects its biological activity.

Our data demonstrate that the reduction in MazEF levels observed in an aMEF mutant strain is substantial enough to affect the biological function of MazEF. A recent report found that Dps levels were affected in a *mazEF* mutant strain after DNA damage (42). We show that *dps* steady-state transcript levels are increased in the aMEF pMUT_F and *mazEF* deletion strains compared to the wild-type strains. These data suggest that MazEF regulates the transcription initiation of *dps* and/or the stability of the *dps* transcript at the exponential growth phase. MazF is an endoribonuclease and could directly degrade the *dps* transcript. In addition, MazE and MazEF have been shown to downregulate the transcription initiation of their own genes and could regulate the transcription initiation of *dps* as well as other genes (59, 60). Cellular levels of Dps are relatively low at the exponential growth phase and increase in response to oxidative stress or entrance into the stationary growth phase (61, 62). We suggest that at the exponential growth phase, MazEF maintains low levels of Dps when it is not required.

### Concluding remarks.

Taken together, our data demonstrate that the transcription of aMEF and/or the aMEF transcript regulates the expression of MazEF. aMEF is lowly expressed and undetectable by Northern blotting in wild-type cells. The aMEF promoter sequence is not well conserved or similar to the canonical σ^70^ promoter in the −10, −35, or extended −10 TGN regions (48). However, RNA polymerase can initiate transcription from diverse DNA sequences, resulting in low levels of genome-wide transcription, termed pervasive transcription. Pervasive transcription has been dismissed as artifacts or nonfunctional transcriptional noise by some (4, 5). We and others have proposed that pervasive transcripts could be functional and beneficial in some instances (3, 49). To our knowledge, this is the first report to demonstrate that, indeed, a pervasive transcript (aMEF) is functional and regulates *mazEF* gene expression in E. coli. Our data indicate that low levels of promoter activity or sequence conservation should not be used to predict a lack of functionality.

Finally, we and others have identified antisense RNAs opposite many type II TA systems; however, this is the first report characterizing their role in gene regulation (4, 28, 29, 63). It is tempting to speculate that asRNAs may be ubiquitous regulators of type II TA systems, but this remains to be determined.

## MATERIALS AND METHODS

### Bacterial strains and growth conditions.

The E. coli strains and oligonucleotides used in this study are listed in Data Set S1 in the supplemental material. Cells were grown in LB at 37°C with aeration (300 rpm) to the logarithmic growth phase, at an optical density at 600 nm (OD_600_) of ∼0.4 to 0.6 unless otherwise specified. The medium was supplemented with kanamycin (15 or 50 μg/mL) when appropriate.

10.1128/mbio.03443-21.6 DATA SET S1 (Sheet 1) Legend; (sheet 2) strains and plasmids; (sheet 3) oligonucleotides and primers (enzyme cut sites are in boldface type); (sheet 4) gene blocks for *mazE* and *mazF* strain construction; (sheet 5) Northern probes. Download Data Set S1, XLSX file, 0.02 MB. Copyright © 2022 Van Gundy et al. 2022 Van Gundy et al. https://creativecommons.org/licenses/by/4.0/ This content is distributed under the terms of the Creative Commons Attribution 4.0 International license.

### GFP reporter construction.

The pWM1015 plasmid with a consensus Campylobacter promoter (pcampy) directly upstream of a *gfp* gene was used as the positive control for constitutive *gfp* transcription, and the parent vector was used for all promoter fusion constructs (30). To generate the negative-control, promoterless construct, the pcampy promoter was removed from pWM1015 by digestion with EcoRI and BamHI (New England BioLabs [NEB]), treated with DNA polymerase Klenow fragment (New England BioLabs), and blunt-end ligated using T4 DNA ligase (New England BioLabs) according to the manufacturer’s instructions. The promoter sequences were generated by annealing complementary oligonucleotides corresponding to the 75 bp upstream of the +1 transcriptional start sites of the respective promoters. The promoter oligonucleotides also included engineered EcoRI and BamHI sites flanking the promoter sequence to allow digestion and insertion into the pWM1015 plasmid. Oligonucleotide annealing was performed by combining 500 pmol of each oligonucleotide in a 50-μL reaction mixture, incubating the mixture at 95°C for 5 min, and then slowly cooling the mixture to room temperature. Both the pWM1015 plasmid and the annealed promoter sequences were digested with EcoRI HF (NEB) and BamHI HF (NEB) at 37°C for 1 h. The digests were then subjected to phenol-chloroform-isoamyl (25:24:1) extraction and ethanol precipitation. The purified DNA was ligated with T4 DNA ligase (NEB) at 23°C overnight. The ligation mixtures were transformed into chemically competent DH5α cells, plated onto kanamycin (50 μg/mL) plates, and incubated overnight at 37°C. Colony PCR, using the pWM1015_F and pWM1015_R primers (Data Set S1), was performed with GoTaq Hotstart polymerase (Promega) according to the manufacturer’s protocol to screen for the correct insertion of the promoter sequences. The *gfp* promoter fusion plasmids were isolated and verified by Sanger sequencing (Genewiz) with the pWM1015_MCS_F sequencing primer (Data Set S1).

### Measurement of transcriptional activity via flow cytometry.

Cells containing the transcriptional *gfp* fusion to the aMEF wild-type (aMEF pWT) and mutant (aMEF pMUT) promoters were grown as indicated above, with an OD_600_ of ∼0.6. A 1-mL sample was centrifuged at 13,000 rpm, washed with 1× phosphate-buffered saline (PBS), centrifuged again, and resuspended in 1× PBS (OD_600_ × 500). This resuspension was used to make a 1:100 dilution of the sample using 1× PBS as the diluent. Samples were placed on ice until the flow machine setup was ready. Samples were analyzed by flow cytometry (Guava easyCyte benchtop flow cytometer). Samples were run at an event rate of 3,000 events per s and gated to remove debris. Flow data were analyzed via the inCyte software package. Fluorescence intensity was normalized by the median fluorescence of each sample.

### Strain construction.

The MazF protein was C-terminally tagged and the MazE protein was N-terminally tagged with a 3×FLAG epitope via gene fusions on the chromosome. The *mazE* and *mazF* 3×FLAG-tagged constructs were generated by amplifying a DNA fragment (gBlocks) synthesized by Integrated DNA Technologies (IDT) using Phusion high-fidelity DNA polymerase (New England BioLabs), using primers *mazE*_mutF_clone_XhoI_AscI and *mazE*_mutR_clone_HindIII (Data Set S1) according to the manufacturer’s protocol. The gBlock DNAs synthesized by IDT (Data Set S1) included the *mazF* gene with the mutated aMEF promoter, a C-terminal 3×FLAG tag, and an engineered AscI site downstream of the 3×FLAG tag followed by 188 nt of the downstream sequence and the *mazE* gene with the mutated aMEF promoter, an N-terminal 3×FLAG tag, and an engineered AscI site downstream of the *mazF* gene followed by 188 nt of the downstream sequence. The amplified 3×FLAG-tagged aMEF mutant DNA was cloned into pGEM-T Easy using the pGEM-T Easy vector system (Promega). The plasmid DNA was sequenced (Genewiz) using M13_F and M13_R provided by Genewiz. An AscI-flanked kanamycin resistance cassette was amplified (Phusion high-fidelity DNA polymerase) from FRT-PGK-gb2-neo-FRT template DNA (Gene Bridges) using primers Kan_F_AscI and Kan_R_AscI (Data Set S1) and cloned into pGEM-T Easy. Both plasmids were digested with AscI (New England BioLabs), electrophoresed on 1% agarose gels, and gel purified using a Qiagen gel purification kit. The kanamycin resistance cassette was ligated into the aMEF mutated 3×FLAG construct using T4 DNA ligase according to the manufacturer’s instructions. The ligation reaction mixture was transformed into chemically competent DH5α cells, plated onto kanamycin (15 μg/mL) plates, and incubated overnight at 37°C. Colony PCR using M13_F and M13_R (Data Set S1) was performed to screen for correct transformants, and purified plasmids were sent to Genewiz for Sanger sequencing with M13_F and M13_R. aMEF pMUT and pWT 3×FLAG plasmid DNAs were digested with XhoI and HindIII, followed by gel purification from 1% agarose gels using a Qiagen gel purification kit according to the manufacturer’s protocol.

The *mazEF* deletion construct was generated by amplifying the kanamycin cassette (FRT-PGK-gb2-neo-FRT) using Phusion high-fidelity DNA polymerase with primers containing 50 bp of sequence flanking the *mazEF* operon (Data Set S1). The PCR product was cloned into pGEM-T Easy. The plasmid was digested with XhoI and HindIII (New England BioLabs), electrophoresed on 1% agarose gels, and gel purified using a Qiagen gel purification kit according to the manufacturer’s instructions.

The Quick and Easy E. coli gene deletion kit (Gene Bridges) was used to generate all strains according to the manufacturer’s protocol. Recombination of the linear constructs was induced using the Red/ET recombination system via l-arabinose (final concentration, 0.35%). Briefly, BW25113 cells were grown to exponential phase and transformed with the pRed/ET (*tet* or *amp*) plasmid by electroporation. Cells were then grown to exponential phase in the presence of tetracycline (3 μg/mL) or ampicillin (50 μg/mL); FRT recombinase was induced for 1 h at 37°C. The cells were prepared for electroporation by washing the cells twice with 10% glycerol and spinning the cells down at 11,000 rpm. The linear constructs were electroporated into electrocompetent cells (2510 electroporator, 1-mm gap, 1,350 V; Eppendorf). The cells recovered in 1 mL of LB at 37°C for 3 h and were then plated onto kanamycin (15 μg/mL) plates and incubated overnight at 37°C. Colonies were restreaked onto kanamycin (15 μg/mL) plates, and colony PCR was performed with primers *mazEF*_KO_screen_F and *mazEF*_KO_screen_R for the Δ*mazEF* strain, *mazE*_F_45 and *mazF* down_302_R for aMEF wild-type and mutant *mazF* 3×FLAG strains, and mazE_up_361_F and mazE_179_R for aMEF wild-type and mutant *mazE* 3×FLAG strains (Data Set S1). PCR products for the wild-type and mutant 3×FLAG strains were Sanger sequenced (Genewiz) with as*mazEF*_KO_seq_F for the mutant 3×FLAG strain and seq_FLAG_F, mazE_up_267_seq_F, and mazF_128_F_seq for both *mazE* and *mazF* wild-type and mutant 3×FLAG strains to verify chromosomal aMEF promoter sequences and 3×FLAG tags, respectively (Data Set S1).

### Growth curves.

Single colonies were selected, and cultures were grown overnight to stationary phase, at an OD_600_ of ∼1.0. A 1:100 dilution was made from the culture grown overnight and aliquoted into a Corning flat-bottom 96-well plate (Corning). OD_550_ readings were collected using the 550-nm filter in a BioTek Synergy HT microplate reader. The plate data were recorded, with shaking at 37°C for 18 h, with reads being taken every 20 min.

### MazF activity assay.

The constructs for the assessment of MazF activity were developed using the Atum DNA 2.0 vector system. PCR was used to generate wild-type untagged and pWT and pMUT *mazF* 3×FLAG-tagged constructs using pd441 primer sets and BW25113, pWT, or pMUT genomic DNA as the template (Data Set S1). PCR products were ligated and transformed via the DNA 2.0 rapid ligation system into pd441SR. The ligation mixtures were transformed into chemically competent DH5α cells, plated onto kanamycin (50 μg/mL) plates, and incubated overnight at 37°C. Colony PCR was performed using the pd441_mcs_F and pd441_mcs_R (Data Set S1) primers and sequenced with pd444_seq_R (Data Set S1) (Genewiz). Sequence-validated plasmids were electroporated into BW25113 electrocompetent cells. Electroporated cells recovered at 37°C for 90 min. The cells were plated onto kanamycin (50 μg/mL) plates and placed at 37°C overnight. Growth curve measurements, as described above, were taken for each of the three strains.

### Western blot assays.

E. coli cell lysates were generated by centrifuging 1 mL of the culture and normalizing to the cell number using the OD_600_. An equal volume of 2× Laemmli buffer was added to the cell lysates. Samples were frozen and stored at −20°C. Samples were thawed on ice, boiled at 95°C for 5 min, fractionated on prepoured 16% Tris-glycine gels (Invitrogen), and transferred overnight to a polyvinylidene difluoride (PVDF) membrane (Amersham Hybond 0.2-μm PVDF) at 4°C at 30 mA. Membranes were placed in blocking buffer (1× PBS, 0.1% Tween 20, and 5% nonfat dried milk) for 1 h and washed three times for 5 min with washing buffer (1× PBS, 0.1% Tween 20). The membranes were then incubated with monoclonal mouse anti-FLAG antibody (1:20,000) (catalog number F3165-2MG; Sigma-Aldrich) for 1 h, washed three times for 5 min in wash buffer, and incubated with monoclonal goat anti-mouse antibody (1:20,000) (catalog number 115-035-008; Jackson Immuno) for 1 h. Membranes were washed three times for 5 min with wash buffer and then placed into 1× PBS to prepare them for visualization. The membranes were developed by chemiluminescence using ECL Prime Western blotting detection (GE Healthcare-Amersham) and developed in the c400 Azure Biosystems imager using Azure imaging software. Monoclonal rabbit anti-GroEL antibody (1:80,000) (catalog number G6532-.5ML; Sigma-Aldrich) and a monoclonal goat anti-rabbit antibody–horseradish peroxidase (HRP) conjugate (1:20,000) (catalog number 12-348; Sigma-Aldrich) were used as the loading controls. The above-described methods were performed in the same manner to immunoblot the loading control. Western blots were quantified with ImageJ using the exposure before any saturation was detected on the image. The densitometry of each band was measured using the ImageJ rectangle-and-plot function. GroEL loading control bands were normalized to the strongest signal. The ratio of protein to normalized GroEL was used to graph data.

### RNA isolation and Northern blotting.

RNA was isolated using a hot-phenol protocol (3). Cultures were mixed with a stop solution (95% ethanol, 5% saturated phenol) in an 8:1 ratio. Cultures were centrifuged at 5,000 × *g* at 4°C for 5 min. The supernatant was removed, and pellets were flash-frozen in liquid N_2_. Cell pellets were resuspended in lysis buffer (10 mM Tris-HCl [pH 8.0], 1 mM EDTA, and 0.5 mg/mL Lysozyme) and SDS (final concentration of 0.1%). The cell lysate was heated at 64°C for 2 min, and 1 M sodium acetate (pH 5.2) was added to a final concentration of 0.1%. An equal volume of water-saturated phenol (pH 4.3; Fisher Scientific) was added, and the samples were heated at 64°C for 6 min, inverting the samples every 40 s. The samples were centrifuged at 14,000 rpm for 10 min. The aqueous layer was transferred into 2-mL heavy phase-lock tubes (5Prime) containing an equal volume of chloroform and centrifuged at 13,000 rpm for 7 min. The aqueous layer was then transferred into a tube containing 3 volumes of ethanol, 0.3 M sodium acetate (pH 5.2), and glycogen for overnight precipitation at −20°C. Samples were precipitated the next day at 14,000 rpm for 45 min at 4°C, washed with 70% ethanol, and centrifuged again for 20 min. Samples were resuspended in RNase-free water. RNA quantification and purity were measured using a NanoDrop spectrophotometer, and integrity was evaluated by running RNA out on a 1% agarose gel. RNA was treated with DNase 1 (Roche). For Northern blotting, 10 to 15 μg of RNA was denatured in 2× RNA load dye (Thermo Fisher), heated to 65°C for 15 min, loaded onto a Novex precast 6% Tris-borate-EDTA (TBE)–urea (8 M) polyacrylamide gel (Thermo Fisher) in 1× TBE, and run (180 V for 45 to 60 min). RNA was electroblotted at room temperature (10 V for 1 h in 0.5× TBE) onto HybondXL membranes (Amersham). The membranes were UV cross-linked (FB-UVXL-1000 UV cross-linker; Fisher Scientific) and probed with the DNA oligonucleotide (Data Set S1) in OligoHyb buffer (Thermo Fisher) according to the manufacturer’s protocol. Oligonucleotide probes were end labeled with [γ-^32^P]ATP (Perkin-Elmer) and T4 polynucleotide kinase (PNK) (New England BioLabs) according to the manufacturers’ instructions. Unincorporated ^32^P was removed using illustra MicroSpin G50 columns (GE Healthcare). Purified probes were heated at 95°C for 5 min before being added to the prehybridizing blots. Blots were hybridized at 42°C, with rotation overnight. Membranes were washed twice for 30 min in wash buffer (2× SSC [1× SSC is 0.15 M NaCl plus 0.015 M sodium citrate]–0.1% SDS). Membranes were placed onto Kodiak BioMax maximum-sensitivity (MS) autoradiography film and placed at −80°C for 1 to 10 days depending on the radiation emission given by each membrane. The film was developed on an AFP imaging developer and scanned using an Epson Expression 10000XL scanner. 5S rRNA was used as the loading control.

### Bacterial promoters.

To assess the conservation of the aMEF promoter across bacteria, *mazF-pemK* sequences were obtained from GenBank for the following species: E. coli (accession number CP064683.1), Leptospira interrogans (accession number CP023985.1), and Salmonella enterica (accession number MT742153.1). Since the aMEF promoter is contained within the *mazF* coding region, the translation align algorithm available in Geneious (v.9.1.8) was used to align the promoter region.

### Data availability.

Flow cytometry data have been deposited in FlowRepository (https://flowrepository.org/id/FR-FCM-Z3WJ; accession number FR-FCM-Z3WJ).
