# Supplementary figures and images for: An Antisense RNA Fine-Tunes Gene Expression of the Type II MazEF Toxin-Antitoxin System
Source: mBio. 2022 Jan 11;13(1):e03443-21. doi: 10.1128/mbio.03443-21 (PMC8749433; doi:10.1128/mbio.03443-21)

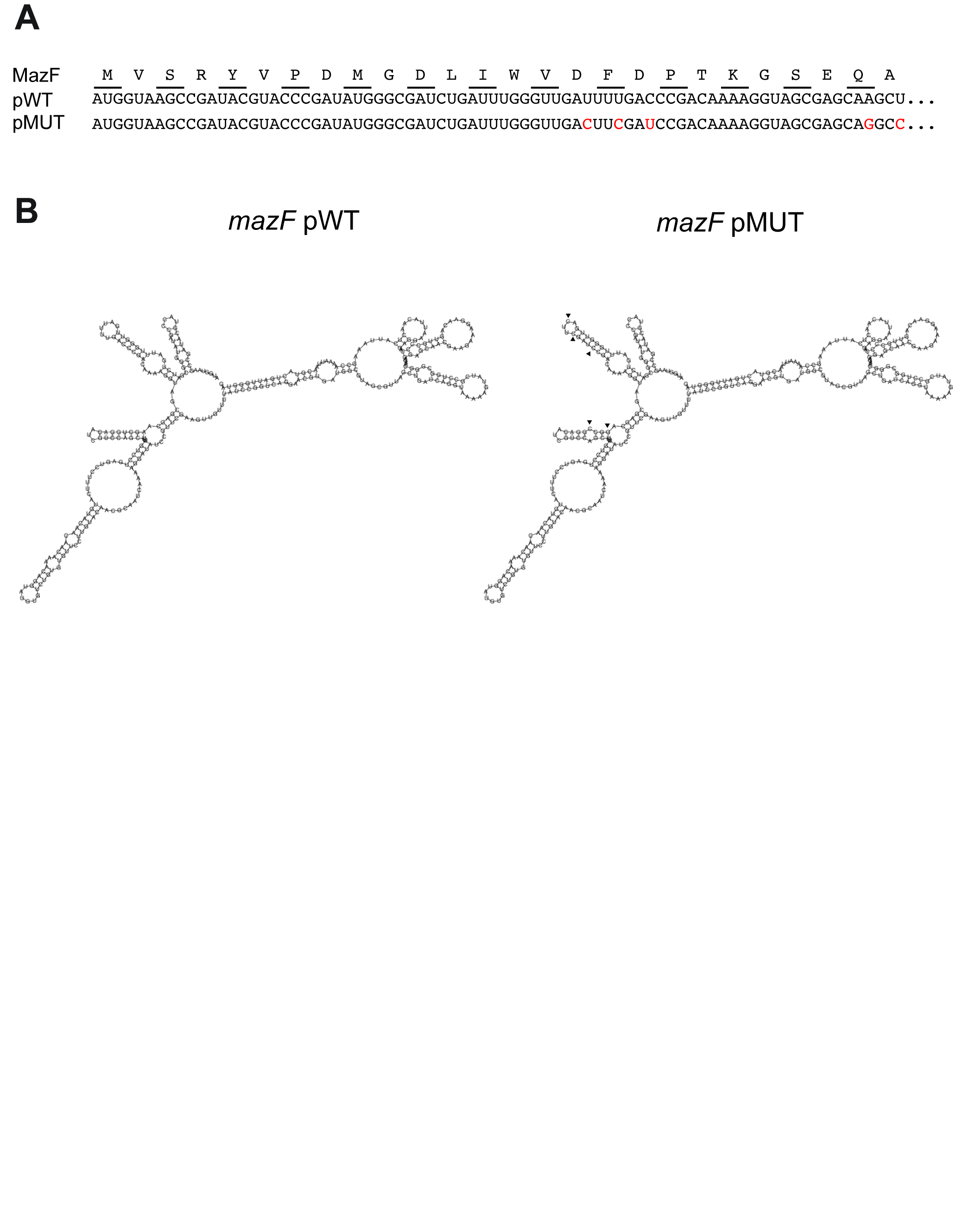

Supplement: FIG S1 [file mbio.03443-21-sf001.tif]

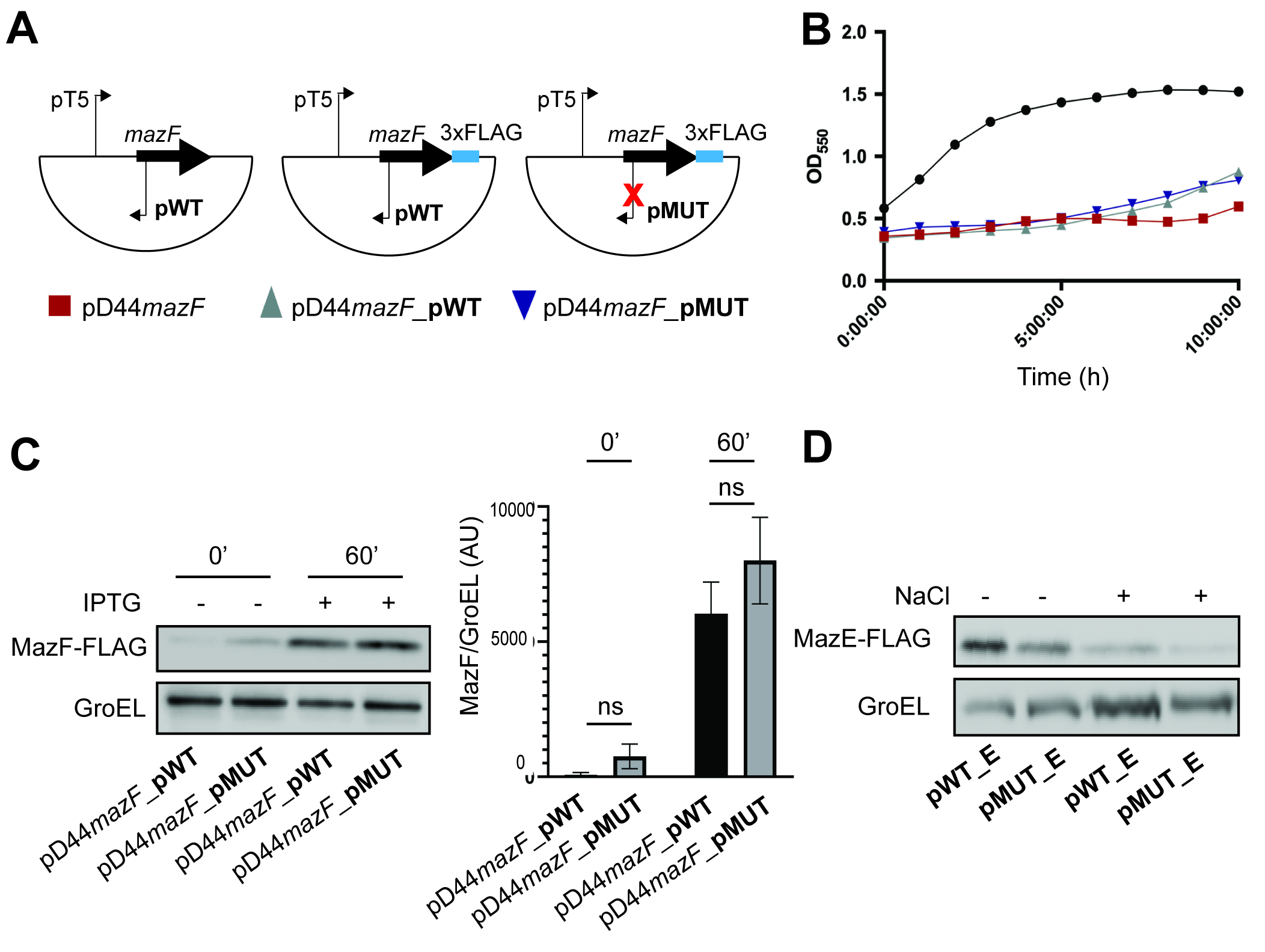

Supplement: FIG S2 [file mbio.03443-21-sf002.tif]

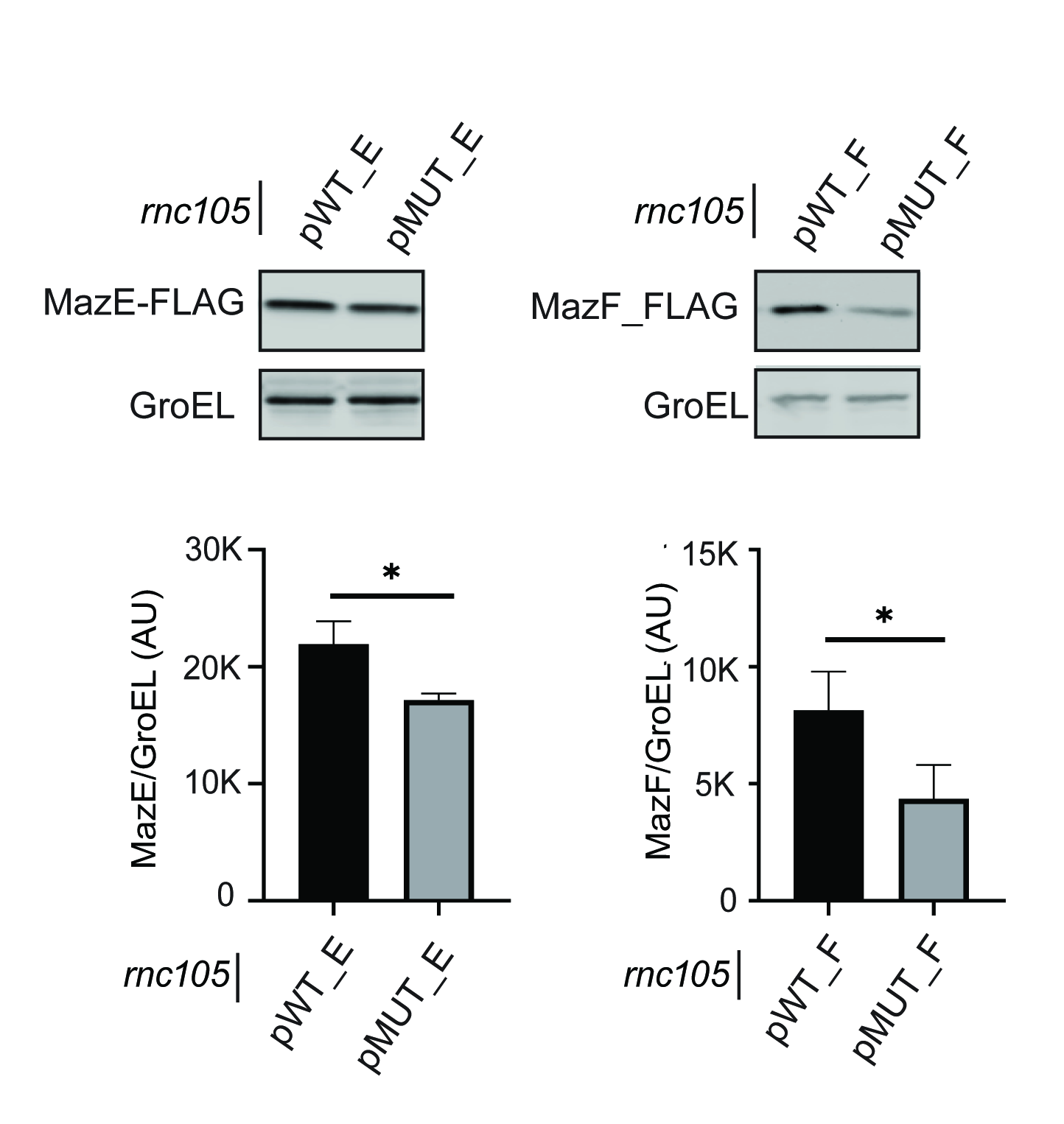

Supplement: FIG S3 [file mbio.03443-21-sf003.tif]

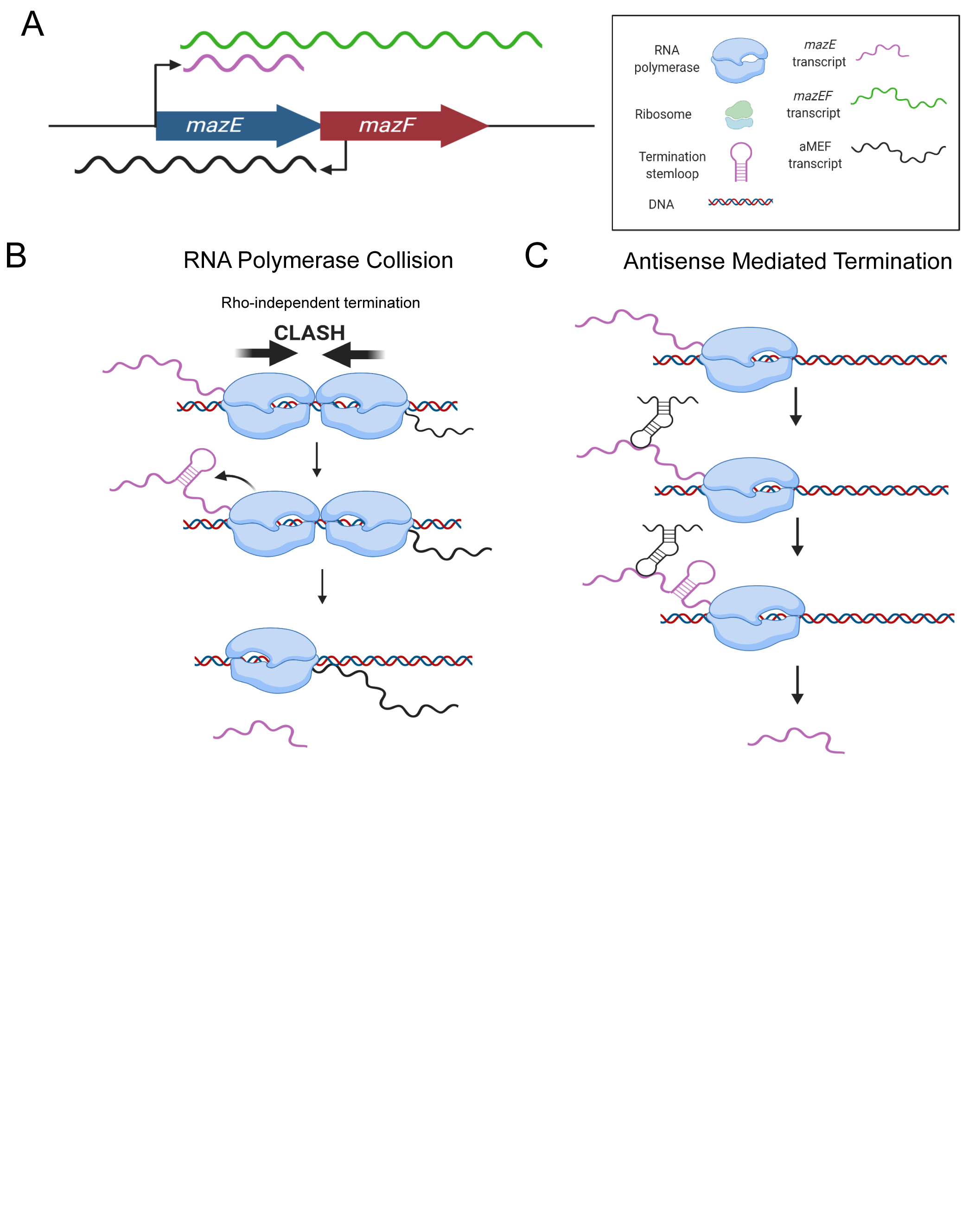

Supplement: FIG S4 [file mbio.03443-21-sf004.tif]

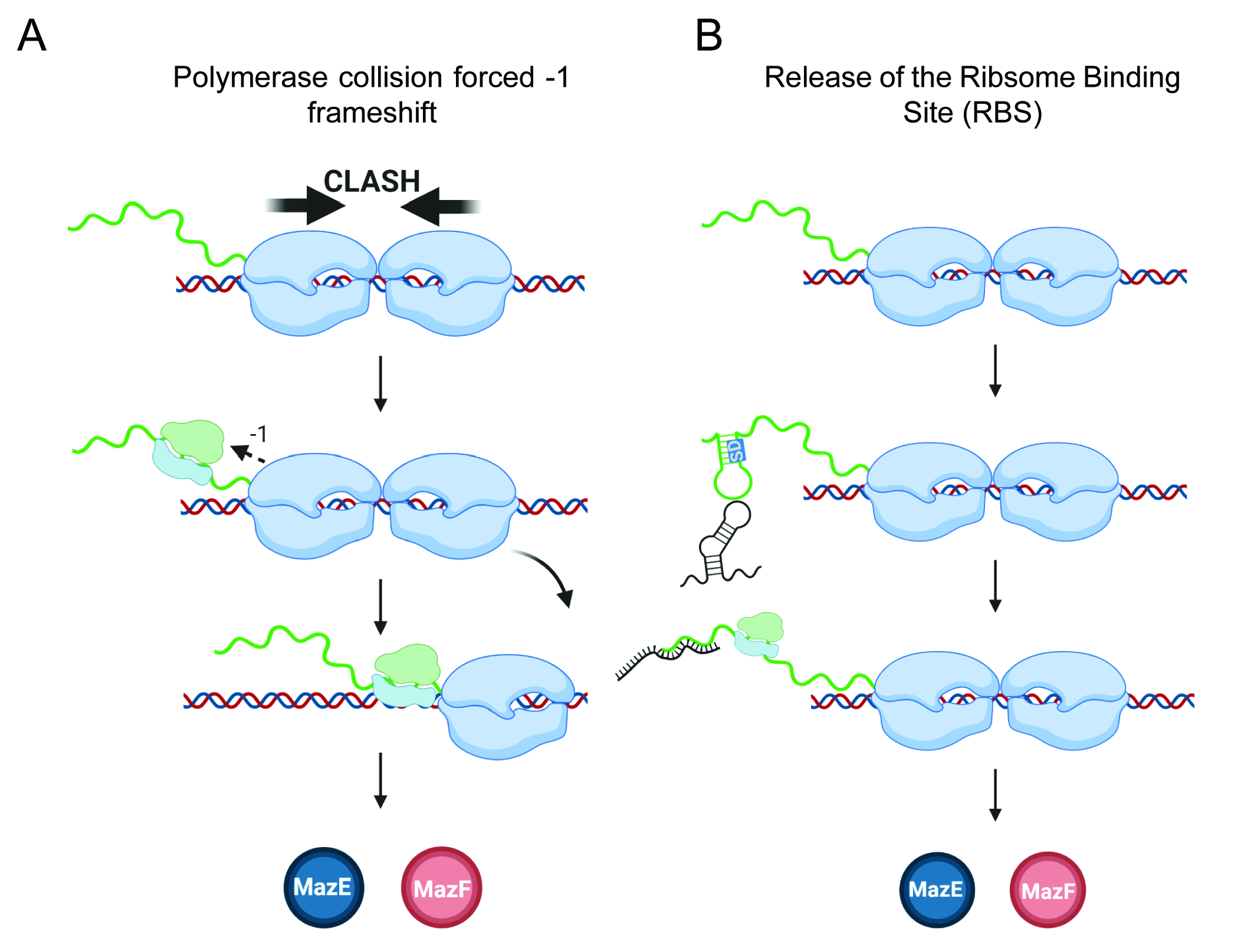

Supplement: FIG S5 [file mbio.03443-21-sf005.tif]
